# A high-saturated, long-chain fatty acid ketogenic diet negatively impacts visual and motor-sensory function in a pre-clinical model of multiple sclerosis

**DOI:** 10.3389/fimmu.2025.1587760

**Published:** 2025-05-22

**Authors:** Erin N. Capper, Jeffrey J. Anders, Benjamin W. Elwood, Randy H. Kardon, Oliver W. Gramlich

**Affiliations:** ^1^ Department of Ophthalmology and Visual Sciences, University of Iowa, Iowa City, IA, United States; ^2^ Center for the Prevention and Treatment of Visual Loss, Iowa City VA Health Care System, Iowa City, IA, United States; ^3^ Department of Neuroscience and Pharmacology, University of Iowa, Iowa City, IA, United States; ^4^ Department of Ophthalmology and Visual Sciences, University of Alabama at Birmingham, Birmingham, AL, United States

**Keywords:** multiple sclerosis, optic neuritis, EAE, ketogenic diet, fatty acids, saturated fats, RGC, electrophysiology

## Abstract

**Introduction:**

Multiple sclerosis (MS) is a neurodegenerative condition that results in demyelination of the central nervous system. Visual impairment, retinal nerve fiber layer thinning, and impaired electrical function in retinal ganglion cells are seen throughout disease progression and serve as useful markers for treatment success. Current research examining the effects of ketogenic diet (KD) as cotherapy show promising anti-inflammatory properties, but research remains limited by differences in experimental set-up and KD composition. The purpose of our study was to use functional and structural biomarkers to determine the neuroprotective effects of a KD composed of long-chain, saturated fatty acids and how the timing of its implementation impacts these biomarkers in an experimental autoimmune encephalomyelitis (EAE) model.

**Methods:**

EAE was induced in 80 female C57BL/6J mice by immunization with MOG35-55 and randomly assigned to stay on the standard diet or to start the KD at one of three time points (preconditioned, prophylactic, or late). Motor-sensory scores, visual acuity, OCT, electrophysiology, and histopathology were performed.

**Results:**

In general, our results show that a KD with long-chain, saturated fatty acids did not significantly improve visual outcomes, and that early implementation of the diet modestly exacerbated motor-sensory and visual acuity deficits despite not impacting optic nerve axonal damage, retinal ganglion cell loss, or psychomotor measurements of visual system function.

**Discussion:**

We propose that the anti-inflammatory neuroprotective benefits of a KD are limited when saturated, long-chain fatty acids are used, and that chain length and fat saturation should be taken into account when utilizing KD as a treatment.

## 1 Introduction

Multiple sclerosis (MS) is a chronic neurodegenerative condition that causes demyelination in the central nervous system (CNS) (1). It is a leading cause of non-traumatic neurological disability in young adults with over 2.5 million cases worldwide (2). In MS, autoimmune demyelination occurs through autoreactive T cells that are inappropriately activated through contact with unidentified antigens (3). The activated lymphocytes transmigrate through the blood brain barrier and cause inflammatory effects in the CNS via cytokine production, leading to symptoms such as pain, motor-sensory disabilities, and visual deficits (4).

Acute optic neuritis (ON), characterized as inflammation of the optic nerve, is the initial presenting symptom in 25% of MS cases and occurs at least once during the disease course in 70% of patients with MS (5, 6). Vision loss from MS-induced ON (MS-ON) is often moderate in nature, with only 35% of patients scoring worse than 20/200 on visual acuity, but many patients continue to have long-lasting disturbances in contrast sensitivity, color vision, depth perception, and visual field deficits (7). Despite initial improvement of visual acuity, patients with MS can continue to have recurring episodes of ON and permanent retinal ganglion cell (RGC) loss characterized by thinning of the retinal nerve fiber layer (RNFL) and RGC layers as measured by optic coherence tomography (OCT) (8). In addition to structural changes, persistent functional alterations can also be detected in patients with MS via pattern electroretinogram (pERG) and pattern visual evoked potential (pVEP) which measure retinal function and optic nerve conduction speed, respectively (9, 10).

Given the inflammatory nature of MS and the contribution of autoreactive T-cells in disease pathogenesis, current MS and MS-ON therapies target immunomodulation (4, 11). While high-dose intravenous corticosteroids are utilized in acute attacks, the first-line treatments of choice for chronic disease are disease-modifying therapies (DMTs) which have been shown to decrease disease progression and relapse by reducing both the number and activation of T-cells (4, 11, 12). Other new therapies that have been explored include plasma exchange which has shown mild short-term visual improvement in patients resistant to high-dose glucocorticoids; however, research is limited by small sample sizes and more information is needed about long-term benefits (13). Other approved therapies include anti-CD20 antibodies such as Rituximab, Ocrelizumab, and Ofatumumab, which are derived from mesenchymal and hematopoietic stem cells (14). While these monoclonal antibodies are used in the treatment of relapsing MS, they have only mild benefits in progressive MS and are associated with a variety of adverse effects like opportunistic infections, secondary autoimmune diseases, and malignancies (15).

Despite advances in pharmacological treatments of MS and MS-ON, long-term outcomes associated with the disease remain poor with ~50% of MS patients experiencing mobility impairment requiring ambulatory aid within 15 years of diagnosis (16) along with persistent RGC loss in a vast proportion of patients regardless of ON history (17). Given the significant morbidity and mortality associated with MS, research investigating the effects of adjuvant and alternative treatments are needed.

A growing body of literature suggests dietary therapies can be effective alternative treatments, and are thought to influence the progression of the disease via production of certain metabolic factors (18). While research has been conducted on various therapeutic diets, the ketogenic diet (KD) has been of particular interest in the treatment of MS (18, 19). Studies looking at the overall effects of KD in MS have shown promising results in attenuating inflammation through reduced expression of enzymes involved in the biosynthesis of proinflammatory molecules; however, research remains limited by differences in experimental set-ups and KD composition (18, 20–22).

In two preclinical studies conducted by Sun et al. and Zhang et al., researchers revealed that implementing a KD in an experimental autoimmune encephalomyelitis (EAE) mouse model resulted in significant reduction in EAE progression along with decreased demyelination, motor deficiency, and microgliosis in the spinal cord (23, 24). Another study by Zyla-Jackson et al. showed that an EAE phenotype was attenuated in mice when given a KD as measured by motor and visual function biomarkers (25). In comparison to significant disease alleviation, research conducted by Kim et al. demonstrated suppression of inflammatory cytokines in EAE mouse brains along with improved motor activity, spatial learning, and memory, but that a KD failed to prevent initial EAE onset (26). Another study by Choi et al. showed that a KD does not appear to reverse EAE progression and resulted in only minor improvements in EAE symptoms. While the diet utilized by Choi et al. failed to mitigate the EAE phenotype, researchers did find that long term safety and feasibility of KD implementation was achievable for patients with MS (27). Upon further analysis of these preclinical studies, differences can be seen in the composition of their KD with Sun et al., Zhang et al., and Zyla-Jackson et al. utilizing medium-chain fatty acid KDs compared to Kim et al. and Choi et al. utilizing long-chain fatty acid KDs. These studies suggest the need for further analysis of the KDs utilized in EAE studies and exploration of how the chain length and saturation effects outcomes.

The purpose of our study was to determine the effects of a KD comprised of long-chain, saturated fatty acids on MS-ON using visual system markers in a MOG-induced EAE model. A long-chain fatty acid KD was chosen due to its proposed ability to prevent hypoxia-inducible factor-1α (HIF-1α) signaling, a pathway whose activation has been linked to MS with recent studies revealing mitigation of MS symptoms upon its inhibition (28, 29). To assess the effects of this KD on inflammatory regulation, three different KD implementation timings were chosen: preconditioned, prophylactic, and late intervention. We hypothesized that the long-chain, saturated KD would have a modest impact on EAE development given former research showing a similar degree of HIF-1α suppression between saturated and unsaturated fats in patient with diabetes (28). Contrary to some of the KD formulations studied in this model (23–25), our study found that a KD diet with long-chain, saturated fatty acids did not significantly improve motor-sensory or visual outcomes in EAE mice, and that implementation prior to disease manifestation resulted in worse motor-sensory deficits and visual acuity.

## 2 Materials and methods

### 2.1 Animals and study design

All animal experiments were approved by the Iowa City IACUC and were conducted in accordance with the ARVO Statement for the Use of Animals in Ophthalmic and Vision Research. Ninety-six female C57BL/6J (B6) mice were obtained from Jackson Laboratories (Bar Harbor, ME, USA). The use of female mice was decided based on the higher prevalence of females within the MS demographic (6). All animals were placed on a 12-hour light-dark cycle (735 lux) with access to water and food *ad libitum*. EAE-ON was induced in 80 mice on day 0 using MOG immunization. The remaining 16 mice did not undergo EAE induction and served as an age-matched naïve cohort. Each of the 4 EAE cohorts was randomly assigned to either remain on the standard mouse diet or to transition to the KD at one of three predetermined start dates. The naïve group was kept on the standard diet. Daily monitoring of motor-sensory impairment was performed to assess disease progression. Visual acuity was measured weekly using optokinetic response (OKR). Structural imaging was performed using OCT at day 21 and at the end of the study for all animals. pERG and pVEP were also performed at the conclusion of the study prior to euthanasia. Assessment of outcome parameters was performed by trained laboratory personnel who were masked to the study groups (EAE and naïve) and treatment assignment (diet type and timing of implementation). In accordance with current AVMA recommendations, all animals were euthanized by CO_2_ inhalation using a flow rate that displaced 30-70% of the chamber volume per minute, followed by cervical dislocation. Tissue was harvested for immunohistochemistry staining.

### 2.2 Dietary treatment

Cohorts of EAE mice (n=20/group) were randomly assigned to either a standard mouse diet or KD obtained from Research Diets. The overall proportion of macromolecules within the selected KD was 10.4% protein, 0.1% carbohydrate, and 89.5% lipid. The specific lipid composition for the selected KD consisted of 46.6% saturated fat, 34.4.% monounsaturated fat, and 19% polyunsaturated fat, with the majority of the fatty acids being long chain fatty acids such as oleic acid (C18:1), stearic acid (C18), palmitic acid (C16:1), and linoleic acid (C18:2). The 3 cohorts of EAE induced mice assigned to the KD were further divided into one of the following pre-determined time intervals for diet implementation: before EAE induction (5 days prior), at the time of EAE induction (day 0), or at the time of EAE symptom onset (approximately day 7). The remaining EAE group and the naïve group remained on the standard mouse diet for the full duration of the study.

### 2.3 EAE-ON model

EAE-ON was induced in eighty 2-month-old female C57BL/6J (B6) mice (The Jackson Laboratories, Bar Harbor, ME) using previously described methods (30, 31). Briefly, mice were induced using a 200 ug solution composed of a 1:1 ratio of MOG_35–55_ peptide (200 µg; Sigma Aldrich, St. Louis, MO, USA) and complete Freund’s adjuvant (Sigma Aldrich) which contained *desiccated Mycobacterium tuberculosis* H37Ra (200 µg; BD Difco, Franklin Lakes NJ, USA). The mixture was injected subcutaneously at two spots along the back. The 80 mice subsequently received a 400ng intraperitoneal injection of pertussis toxin mixed with PBS at day 0 and again on day 2. Daily motor-sensory function was assessed by a trained investigator using a 5-point EAE scale with the following criteria: 0 = no symptoms, 0.5 = partial tail paralysis, 1 = tail paralysis, 1.5 = partial tail paralysis and waddling gait, 2 = tail paralysis and waddling gait, 2.5 = partial limb paralysis, 3 = paralysis of one limb, 3.5 = paralysis of one limb and partial paralysis of another, 4 = paralysis of two limbs, 4.5 = moribund state, 5 = death. EAE was induced in five different batches (n=5/EAE group/day) of randomly assigned mice.

### 2.4 Visual acuity

Bilateral visual acuity was assessed in each mouse using the OptoDrum system (Striatech, Tübingen, Germany) as previously described (32). Cohorts were measured at baseline and then in 7-day intervals throughout the duration of the study. Awake mice were placed on the platform within the closed chamber and visual stimuli were displayed on the 4 monitors composing the chamber walls. The stimuli consisted of cylindrical projecting stripes with a fixed contrast of 99.8% and varying spatial frequencies. An overhead camera recorded the mice while automated software was used to determine if there was reflexive head movement present. Trials of increasingly small stripe width were performed until a lack of reflexive head movement was seen, indicating the threshold of visual acuity of that animal. The last perceived spatial frequency was recorded as cycles/degree (c/d) for each eye.

### 2.5 Electrophysiology

At the conclusion of the study, pERG and pVEP were recorded for all mice using a Celeris platform (Diagnosys, Lowell, MA, USA). Prior to starting, mice were dark-adapted overnight then anesthetized with intraperitoneal injection of ketamine (30 mg/kg, Mylan, Canonsburg, PA, USA), xylazine (5 mg/kg, Akorn Inc., Lake Forest, IL, USA), and acepromazine (2.3 mg/kg, Rattlesnake Drugs, Scottsdale, AZ, USA). Pupil dilation was achieved using 1% tropicamide (Alcon Laboratories, Fort Worth, TX, USA), and GenTeal gel (Alcon Laboratories) was placed on the cornea to preserve corneal integrity. Before and after data collection, animals were kept on heating pads to maintain a constant body temperature of 37°C. pERG/pVEP was evoked using a 2 Hz alternating black and white vertical stimuli at 100% contrast and luminescence of 50 candela/m^2^. A total of 600 traces were performed for each eye and then averaged to create one waveform. Amplitudes were measured in microvolts (µV) from the P1 peak to the N2 trough as a representation of RGC electrical function (9). Latencies were recorded in milliseconds (ms) using the elapsed time from stimulus presentation to the N1 trough which serves as a representation of optic nerve conduction speed (10). Data from eyes with flat or atypical pVEP waveforms or non-unique peak identification were excluded from the statistical analysis.

### 2.6 OCT imaging

OCT was used to analyze the retina and assess for changes in thickness of the retinal nerve fiber layer (RNFL) as well as the retinal ganglion cell (RGC) complex. Mice were anesthetized using a SomnoSuite anesthesia system (Kent Scientific, Torrington, CT, USA) which delivered 2% isoflurane (Baxter Healthcare, Deerfield, IL, USA) at a rate of 498 mL/min within the concealed chamber. The mouse was then transferred to a small platform and continued to receive 1.3% isoflurane (Baxter Healthcare Co.) through a nosecone. Pupils were dilated using 1% tropicamide (Alcon Laboratories) and images were obtained using the SD-OCT instrument (Bioptigen, Morrisville, NC, USA). Measurements of the retinal layers were performed manually between the borders of the RNFL from the inner limiting membrane and the outer boarders of the inner plexiform layer (IPL) in the superior, inferior, nasal, and temporal quadrant at a distance of 400 microns from the optic nerve head. Measurements were taken at the baseline, midpoint, and endpoint of the study. The thickness of the RNFL and RGC complex was calculated for each mouse using an average between the two eyes, and statistics were used to compare the overall change in thickness between the EAE and naïve cohorts. Data from eyes with indistinguishable retinal layer boarders were excluded from analysis.

### 2.7 Optic nerve histopathology

Optic nerves were harvested from all mice at the conclusion of the study and fixed in 2% glutaraldehyde 2% paraformaldehyde phosphate buffer in flat bottomed glass vials. Samples were then prepared for cross-section imaging using a modified TEM embedding protocol (30). Briefly, optic nerves were fixed with 1% osmium, dehydrated in an ethanol series, penetrated with a series of propylene oxide (Sigma 110205)/Eponate 812 resin (18010, Ted Pella Inc., Redding, CA, USA), then embedded in Eponate 812 resin. Semi-thin 500 nm axial sections were cut using a speed of 0.6 mm/s on a Leica EM UC7 Ultramicrotome. The cut samples were transferred to slides and stained with 1% paraphenylenediamine (PPD; Sigma P6001). Imaging was performed at 40x magnification using the Zeiss Axioscope 5 to score optic nerve damage. A standardized 0–5 grading scale was used as follows: grade 0 = healthy optic nerve; 1 = healthy nerve with only a few deeply stained (damaged axons); 2 = slight damage with only a few damaged axons; 3 = moderate damage with frequent degenerated axons; 4 = severe damage with mostly degenerated axons and the appearance of gliotic area; 5 = severe axon degeneration in the optic nerve with frequent gliotic areas (33). Next, optic nerve axon density was determined for each sample using 100x magnification and an eight-micrograph layout resembling a cruciform pattern. Axon counts were completed in a semi-automatic manner using ImageJ plugin AxoNet, as previously described (34). Briefly, TIFF images were opened in ImageJ and areas of interest were scaled to 15.7 pixels/um. The system identified areas of remaining myelinated axons and counted the total number using an algorithm based on U-Net-based encoder/decoder architecture (35). Completed images were manually examined for any obvious discrepancies in the number or position of counted axons. The number of myelinated axons per image was then normalized to the area and were displayed as optic nerve axon numbers relative to the area being sampled (density/mm^2^).

### 2.8 Statistics

All data were assessed by trained personnel who were masked to animal and treatment groups. Data for EAE scoring was performed using area under the curve (AUC) to compare overall disease severity between cohorts. OKR, OCT, electrophysiology, and histopathology data were analyzed using two tests after confirming normal distribution using Shapiro- Wilk testing. For numeric data, a one-way ANOVA was used to compare all EAE cohorts to the naïve to determine if the mouse model worked. Next, a Tukey *post hoc* analysis was performed to determine if there was significant difference between the standard diet EAE cohort and the other three cohorts assigned to KD. For ordinal data, a Kruskal- Wallis test for multiple comparisons was used. All calculations were performed using GraphPad Prism v9.4 (GraphPad Software, San Diego, CA, USA), and p-values less than 0.05 were considered significant. Only significant differences between cohorts are shown in the graphs.

## 3 Results

### 3.1 Early implementation of KD worsens motor-sensory deficits in EAE mice

**Figure 1:**
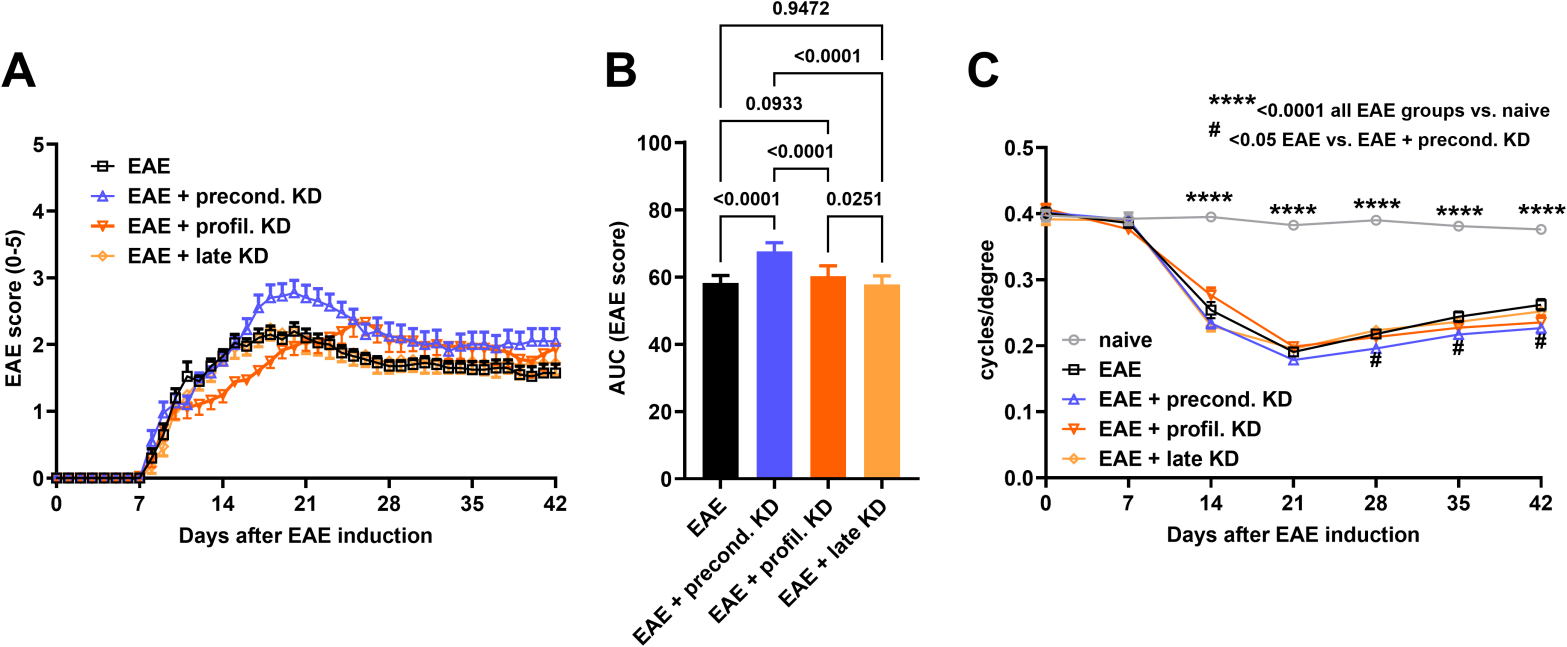
Daily EAE score analysis to assess motor-sensory deficits: **(A)** Average EAE score for each cohort (n = 20/group) is displayed throughout the duration of the study. There is a significantly lower EAE score at day 14 in the EAE prophylactic KD cohort when compared to standard diet EAE mice. In contrast, the EAE preconditioned KD cohort had significantly higher EAE scores at day 21 when compared to the EAE standard diet group. **(B)** EAE score analysis using AUC demonstrates a significant increase in motor-sensory impairment in the EAE preconditioned KD group (n = 20) when compared to the EAE prophylactic KD (n = 20), EAE late KD (n = 20), and EAE standard diet mice (n = 20). **(C)** All four EAE induced cohorts exhibit significantly worse visual acuity when compared to naïve control (n =16) starting at day 14. In addition, the EAE preconditioned KD cohort shows significantly worse visual acuity compared to the EAE standard diet group starting at day 28 through the end of the study. All data are shown as Mean ± SEM. **** <0.0001 all EAE groups vs. Naive; # <0.05 EAE vs. EAE + precond. KD.

Average daily EAE score was plotted for each cohort to assess disease progression. All EAE-induced animals displayed a monophasic disease course with the first clinical symptoms presenting at approximately day 7 (**Figure 1A**). Symptoms presented with increasing severity in a caudal-to-rostral pattern. Motor-sensory scores peaked around day 21 in all EAE mice except for the EAE prophylactic KD cohort which peaked around day 28. Significantly lower EAE scores were seen for the EAE prophylactic KD cohort around day 14. Significantly higher EAE scores were seen for the EAE preconditioned KD cohort through days 20–28 when compared to the EAE standard diet. Throughout the 42-day time course, the EAE preconditioned KD cohort displayed the worse overall progression when compared to the other EAE treated cohorts (AUC EAE score: EAE: 58 ± 2; precond. KD: 68 ± 3, p<0.001; profil. KD: 60 ± 3, p=0.09; late KD: 58 ± 3). All other EAE KD cohorts had no significant difference in motor deficits from the EAE standard diet cohort (**Figure 1B**).

### 3.2 Early implementation of KD worsens visual acuity in EAE mice

Average visual acuity for each cohort was measured at baseline prior to EAE-ON induction and then weekly thereafter. Results of visual acuity followed a similar trend as the clinical EAE scores. Overall visual acuity remained stable throughout the duration of the study with an average of 0.38 ± 0.03 c/d for naïve control mice. All EAE induced mice had a significantly worse visual acuity when compared to the naïve cohort (p<0.0001, **Figure 1C**) starting at day 14. Another significant difference was seen between the EAE preconditioned KD group (0.23 ± 0.05 c/d) when compared to the EAE standard diet cohort (0.26 ± 0.05 c/d, p=0.024) beginning at day 28 until the end of the study. There was no significant difference between the EAE standard diet mice compared to the EAE prophylactic (0.24 ± 0.06 c/d) and late (0.25 ± 0.05 c/d) KD mice.

### 3.3 KD does not mitigate or worsen RGC complex thinning

**Figure 2:**
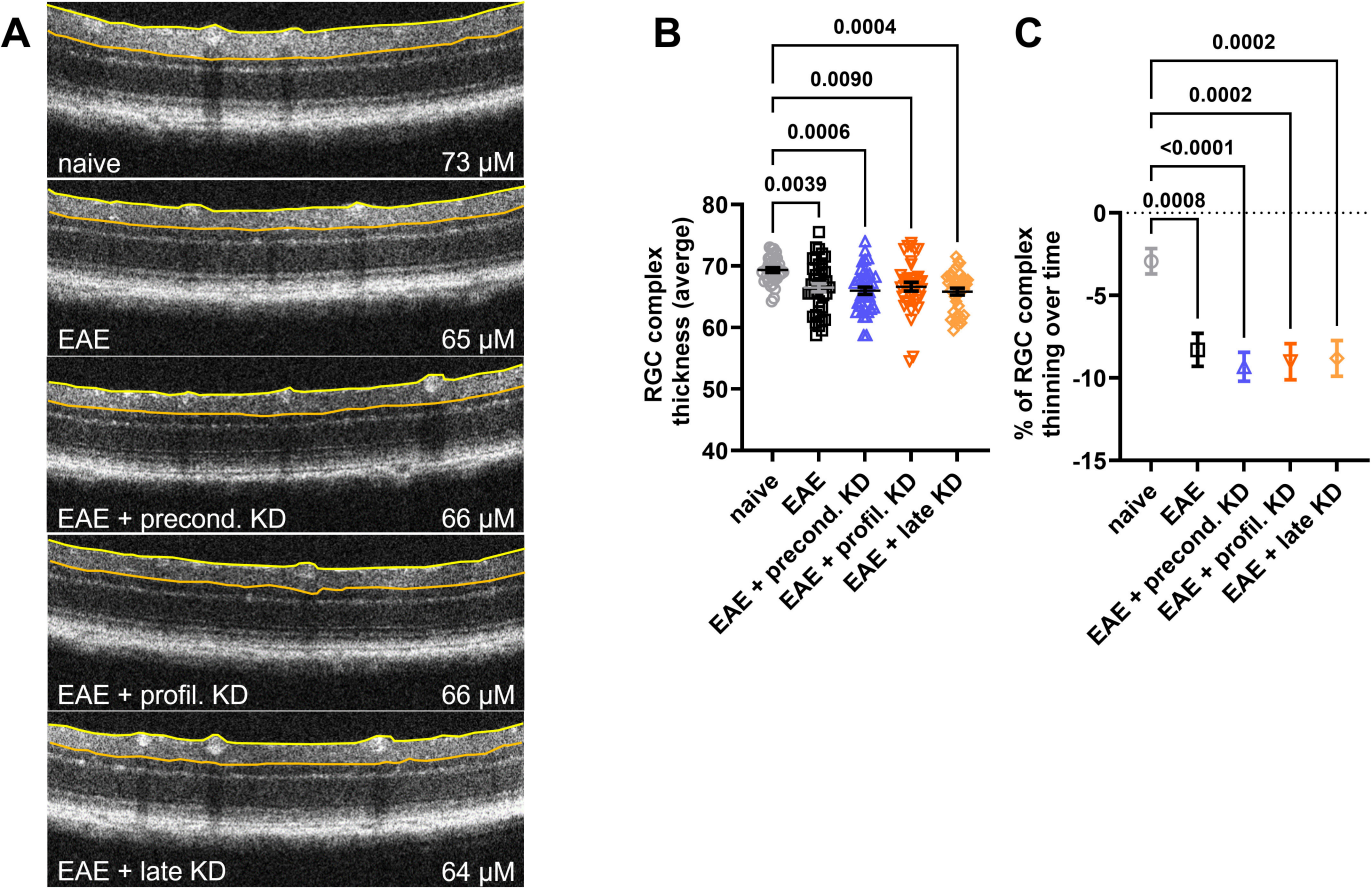
RGC Complex thickness analysis at day 42: **(A)** RGC Complex thickness was measured between the edge of the retinal nerve fiber layer (yellow line) and the border of the inner plexiform layer (orange line). Representative OCT images demonstrate thinning of the RGC complex in all EAE animals. **(B)** Measurements at day 42 revealed that the RGC complex was significantly thinner in all EAE cohorts when compared to naïve controls. There was no difference in thickness between the EAE mice on KD vs. standard diet. **(C)** Percentage of RGC complex thinning between the EAE induction and the 42-day end timepoint was significant in EAE cohorts when compared to naïve controls. Again, there was no significant difference in the percent thinning between the EAE mice on KD vs. standard diet. All data are shown as Mean ± SEM.

OCT was used to measure the RGC/IPL complex thickness as degeneration within the retinal layers are known to be associated with declining retinal function and disease progression in MS patients (15). Images were obtained in the superior, inferior, temporal, and nasal quadrants 400 microns from the optic nerve head. Trained personnel utilized a digital caliper to measure the distance between the top of the RNFL layer and the bottom of the RGC/IPL complex (**Figure 2A**). Baseline OCT imaging did not reveal abnormal retinal pathology in any mouse that would necessitate exclusion from subsequent RGC complex thickness analysis. At day 42, results showed significantly lower RGC complex thickness in all EAE animals when compared to the naïve controls. No significant differences were seen between the four EAE cohorts (**Figure 2B**). This aligned with the analysis of RGC complex thinning, which revealed significantly greater thinning over time in all four EAE induced mice when compared to the naïve control, but no difference in thinning between the EAE preconditioned KD, EAE prophylactic KD, EAE late KD, and EAE standard diet mice (**Figure 2C**).

### 3.4 KD has no effect on pERG and pVEP in EAE mice

**Figure 3:**
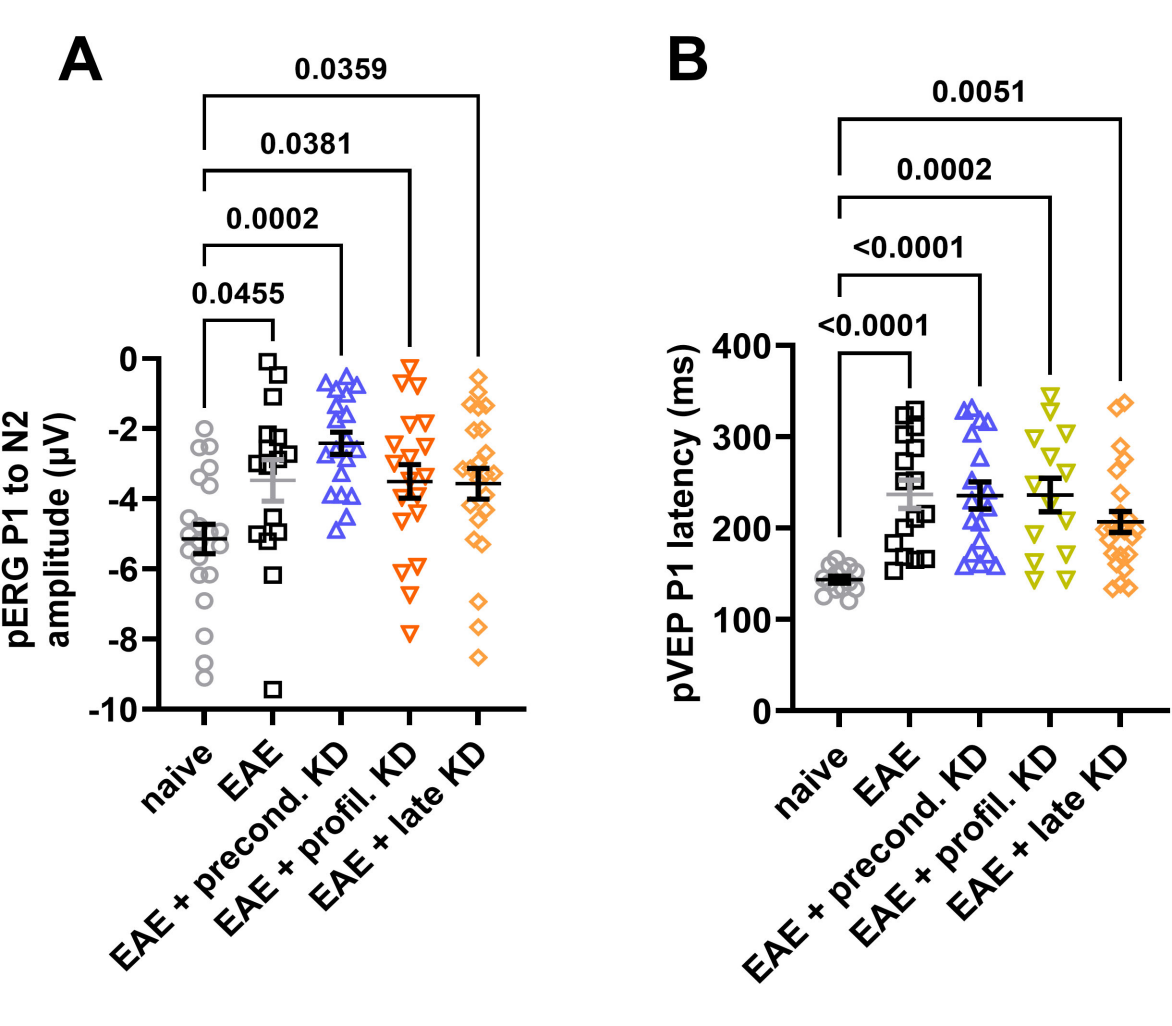
Visual system function measured by pERG and pVEP: **(A)** End of study analysis demonstrates a significant decline in pERG amplitude in all EAE cohorts compared to the naïve control group. No significant differences were detected between the EAE cohorts on KD and standard diet. **(B)** All EAE animals show a significant increase in P1 latency compared to naïve controls. There was no significant difference between EAE cohorts. All data are shown as Mean ± SEM.

pERG and pVEP were recorded to assess RGC and optic nerve function, respectively. Measurements for pERG assessed the difference in the P1 to N2 waveform. This serves as a marker of RGC function, as it is directly related to the response of the cells when exposed to the alternating stimulus (9). Data analysis showed that the total amplitude was significantly lower for all EAE induced mice compared to the naïve cohort. No significant differences in amplitude were detected between any of the EAE cohorts (**Figure 3A**). For pVEP, the time delay between stimulus presentation and the P2 peak was measured, which represents the time it takes for the visual stimulus to travel from the retina to the visual cortex (10). Similar to the pERG data, all four EAE cohorts displayed an increased latency when compared to the naïve cohort, but there was no significant difference in latency between than four EAE cohorts (**Figure 3B**).

### 3.5 KD has no effect on neurodegeneration in optic nerve histology

**Figure 4:**
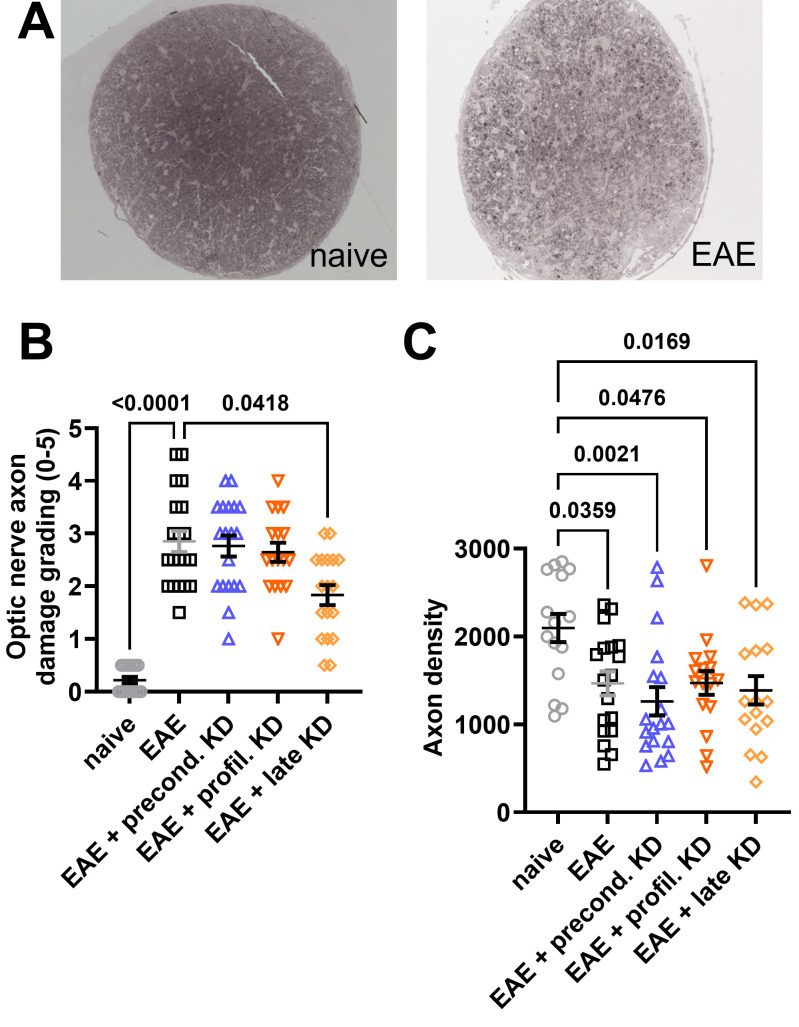
Optic nerve cross section histopathology: **(A)** EAE optic nerves stained with paraphenylenediamine (PPD) reveal increased optic nerve damage and decreased axon density when compared to naïve mice. **(B)** All EAE cohorts display significantly increased optic nerve damage when compared to the naïve controls. However, implementation of KD after symptom onset (EAE late KD) was associated with significantly decreased optic nerve damage compared to the EAE standard diet. There was no difference in axon damage between the EAE standard diet, EAE preconditioned KD, and EAE prophylactic KD cohorts. **(C)** All EAE cohorts has significantly lower axon counts compared to the naïve control group. No difference in axon count was observed between EAE mice on KD vs. standard diet. All data are shown as Mean ± SEM.

After tissue was harvested at the endpoint of the study (day 42), neurodegeneration was assessed via histopathology. The axon density and degree of optic nerve damage was determined using axial sections. For each mouse, one optic nerve was stained and visualized with PPD (**Figure 4A**). The degree of demyelination was graded 0–5 using previously established criteria (33). The images were then analyzed using ImageJ plugin AxoNet to determine the axon count. Results showed significantly worse optic nerve axon damage in all EAE mice when compared to the naïve cohort. However, there was significantly less axon damage in the EAE late KD cohort when compared to the EAE standard diet cohort (p=0.0418, **Figure 4B**). Axon counts showed that all EAE induced mice had significantly lower axon counts compared to the naïve group (**Figure 4C**). There was no significant difference in axon count between the four EAE cohorts.

## 4 Discussion

KDs are high fat, low carbohydrate diets that lead to the production and utilization of ketone bodies. The potential mechanisms of KDs and ketone bodies are multifaceted, and preclinical research indicates potential anti-inflammatory properties that may be beneficial in the treatment of MS. However, evidence remains limited by variations in experimental set up and KD composition (18, 21, 36, 37). The purpose of our study was to determine whether a KD comprised of long-chain, saturated fatty acids shows efficacy in an animal model of MS-ON. Our results demonstrate that irrespective of the timing of its implementation, a long-chain, saturated fatty acid KD does not significantly improve visual outcomes in EAE mice. Furthermore, implementation of the diet before disease onset modestly worsened motor-sensory deficits and visual acuity but did not cause an accompanying exacerbation of optic nerve axonal damage, loss of RGCs, or deficits in pERGs or pVEPs.

It is well known that MS results in motor and sensory deficits leading to significant impairment in mobility (16, 30). The EAE mouse model chosen for our study has well established motor deficit scoring that is a proven indicator of disease severity, meaning that successful disease induction in the mice can be established (38). When assessing motor-sensory scores for the EAE standard diet cohort versus the naïve cohort, there was a significant increase in EAE score severity suggesting that an adequate MS phenotype was induced. Further analysis of EAE scores revealed that all EAE mice had a monophasic disease course with peak symptoms on day 21, except for the EAE prophylactic KD group which had a delayed peak of symptoms to day 28. Although the EAE prophylactic KD group had significantly lower EAE scores than the EAE standard diet at days 14-17, the AUC had no significant difference leading us to conclude that the lower EAE scores is not due to actual protection from KD but that the normal time course of the disease was delayed for the group. With this taken into account, the analysis of the data as a whole revealed that there was no mitigation of visual outcomes other than slightly reduced optic nerve damage in the EAE late KD cohort, and that preconditioned implementation of KD worsened motor-sensory and visual acuity outcomes.

When comparing these results to other literature, we found that our findings varied from most of the prior research conducted examining the effects of a KD in the EAE mouse model (23–25). We propose that the likely reason for these differences in EAE severity and neuroprotection is due to the composition of the KD being used. In the aforementioned study by Zyla-Jackson et al., researchers were some of the first to analyze the specific composition of their KD which was composed of medium-chain fatty acids and poly-unsaturated fatty acids (omega-3). The results of this low-saturated, medium chain fatty acid diet were almost a complete amelioration of the EAE motor and visual deficits (25). Around the same time, Sun et al. and Zhang et al. released studies also examining the effects of a medium-chain fatty acid KD which suggested similar improvements in inflammation and neuroprotection in the EAE phenotype (23, 24). On the other hand, the KD utilized in the above studies by Kim et al. and Choi et al. was a modified, high fat diet from Bio-Serv that was composed of linoleic (C18:2) and linolenic acid (C18:3), both of which are long chain fatty acids (26, 27). The KD used in our own study was obtained from Research Diets and consisted of multiple long chain fatty acids, the majority being oleic acid (C18:1) followed by stearic acid (C18), palmitic acid (C16:1), and linoleic acid (C18:2). Additionally, the diet’s total fat composition consisted of 46.6% saturated fat, 34.4.% monounsaturated fat, and 19% polyunsaturated fat.

Based on the composition of these KDs, we propose that having a KD high in saturated, long-chain fatty acids is not beneficial at remediating the EAE disease course or its associated motor and visual symptoms. Although we can only speculate, we propose that pretreatment with a long-chain, saturated KD, as seen in the preconditioned KD cohort, leads to an initial priming of the immune system that results in a more robust immune response and inflammatory cascade when disease induction occurs. Previous research examining the effects of a highly saturated fatty diet, not administered as part of a KD, in patients with MS has been shown to induce inflammatory cascades and lead to gut microbiome disruption (39). This effect is thought to occur because of the ability of the saturated fatty acid to mimic the actions of lipopolysaccharide, a molecule that stimulates innate immune cells leading to subsequent inflammation (40). What has been studied less extensively is the effect of fatty acid chain length in the role of inflammation. Research looking at the anti-inflammatory properties of short-chain and medium-chain fatty acids has shown promising effects (25, 41). Proposed reasons for their greater therapeutic effects include differences in fatty acid transportation during uptake, such as the use of monocarboxylate transporter receptors, and the ability for direct absorption into portal circulation compared to the need for chylomicrons and lymphatic system passage for long chain fatty acids (41, 42). Compared to short-chain and medium-chain fatty acids, the pro-inflammatory versus anti-inflammatory properties of long-chain fatty acids remains controversial with more research needed to understand exactly what properties of these fats alter inflammatory gene expression (43).

While our study suggests a potential link between KD composition and degree of neuroprotection in an animal model of MS, one limitation of the study is that we did not directly compare a KD with a high saturated, long-chain fatty acid composition compared to one with an unsaturated, long-chain fatty acid composition. Testing this comparison in the future will be useful to determine whether saturation or chain-length is more important to reducing inflammation in neuroinflammatory conditions. Another limitation of our study is that it took place over the course of 42 days. The duration of clinical studies tends to range from a minimum to 2 to 3 weeks up to 6 to 12 months (44), our study being on the shorter end makes it hard to relate our findings with long term outcomes of MS. On the other hand, strengths of our study include a large sample size with highly translational visual system measurements that can be directly translated to clinical use for future clinical trials (45).

To summarize, MS is a chronic neurodegenerative disease that is associated with ON and other long-lasting visual deficits (1, 4). Despite current therapies, patients with MS continue to experience high rates of motor and vision related morbidities, thus the need for development of alternative and adjuvant therapies (7). While there is growing evidence of anti-inflammatory benefits of KD therapy in MS (18, 20, 21), we present new data that the use of KD in the treatment of MS should take the composition of the KD into consideration.

## Data Availability

The raw data supporting the conclusions of this article will be made available by the authors, without undue reservation.
